# Immune-endocrine network in diabetes-tuberculosis nexus: does latent tuberculosis infection confer protection against meta-inflammation and insulin resistance?

**DOI:** 10.3389/fendo.2024.1303338

**Published:** 2024-01-24

**Authors:** Vivekanandhan Aravindhan, Srinivasan Yuvaraj

**Affiliations:** Department of Genetics, Dr Arcot Lakshmanasamy Mudaliyar Post Graduate Institute of Basic Medical Sciences (Dr ALM PG IBMS), University of Madras, Chennai, India

**Keywords:** latent tuberculosis, insulin resistance, diabetes, tuberculosis, cytokines, hormones, synergy, antagonism

## Abstract

Tuberculosis patients with diabetes, have higher sputum bacillary load, delayed sputum conversion, higher rates of drug resistance, higher lung cavitary involvement and extra-pulmonary TB infection, which is called as “Diabetes-Tuberculosis Nexus”. However, recently we have shown a reciprocal relationship between latent tuberculosis infection and insulin resistance, which has not been reported before. In this review, we would first discuss about the immune-endocrine network, which operates during pre-diabetes and incipient diabetes and how it confers protection against LTBI. The ability of IR to augment anti-TB immunity and the immunomodulatory effect of LTBI to quench IR were discussed, under IR-LTB antagonism. The ability of diabetes to impair anti-TB immunity and ability of active TB to worsen glycemic control, were discussed under “Diabetes-Tuberculosis Synergy”. The concept of “Fighter Genes” and how they confer protection against TB but susceptibility to IR was elaborated. Finally, we conclude with an evolutionary perspective about how IR and LTBI co-evolved in endemic zones, and have explained the molecular basis of “IR-LTB” Antagonism” and “DM-TB Synergy”, from an evolutionary perspective.

## Introduction

**Figure 1:**
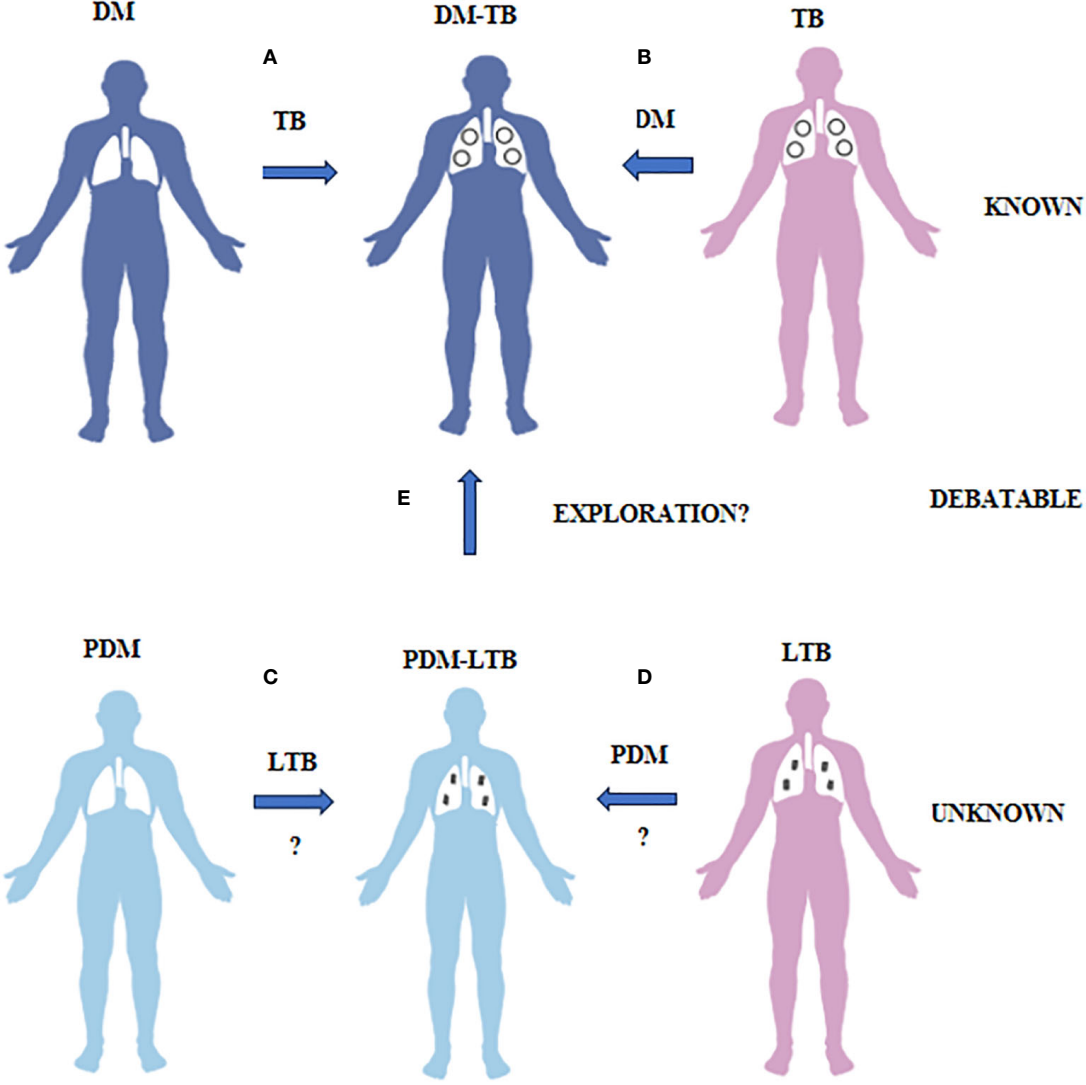
What is known, unknown and debatable in diabetes-tuberculosis synergy and prediabetes-latent tuberculosis antagonism is illustrated. **(A)** Diabetes patients have increased risk of developing TB due to impaired immunity. **(B)** TB patients have increased risk of developing diabetes both due to infection and treatment. This phenomenon is called as DM-TB synergy and is known. **(C)** Prediabetes subjects have increased resistance to TB infection due to insulin resistance. **(D)** LTB subjects have increased resistance to diabetes due to TB mediated immunomodulation. This phenomenon is called as PDM-LTB antagonism and is unknown. **(E)** Whether PDM infected with LTB can convert to DM-TB needs exploration.

Worldwide epidemiological studies have clearly shown increased susceptibility of diabetic patients to tuberculosis (TB), which is often called as “Diabetes-Tuberculosis Synergy” (1, 2) (**Figure 1**). TB patients with diabetes, have higher sputum bacillary load, delayed sputum conversion, and higher rates of drug resistance (1, 2). They also show higher lung cavitary involvement and extra-pulmonary TB infection, which is difficult to treat (1, 2). These results imply that, patients with TB and diabetes, may be more seriously ill, and may pose higher risk for spread, in the community (1, 2). Recent epidemiological surveys have clearly shown the possibility of diabetes-tuberculosis (DM-TB) nexus in near future, which needs immediate attention (1, 2). Chronic inflammation has long been identified as a major risk-factor for diabetes (3, 4), which is low-grade, systemic and non-antigen specific, in nature (3, 4) and is often called as meta-inflammation (5). Most importantly, meta-inflammation impairs immunity and makes diabetic subjects more prone for TB infection (3, 4). Diabetic patients also have increased redox stress, which fuels meta-inflammation, and impairs immunity (6). Diabetes in Asian Indians is characterized by younger age of onset, lower body mass index, propensity for central obesity, increased fat/muscle ratio (sarcopenic obesity) and increased insulin resistance (compared to other ethnic populations), which has been described as the “Asian-Indian Phenotype” (7). Whether the Asian-Indian phenotype (which is not fully characterized), is also a major risk factor for TB is not known.

The substantial increase in diabetes, is taking place in the world, where approximately one-third of the population is latently infected with *Mycobacterium tuberculosis* (latent tuberculosis infection-LTBI) (8). TB remains a leading cause of morbidity and mortality due to infection, worldwide (9). Despite years of research, no vaccine is currently available which can confer protection against pulmonary TB, in adults (10). Further, latency, the hallmark of TB infection remains poorly understood (11). In vast majority of infected people, an effective adaptive immune response develops which controls the replication of the bacteria but is not sufficient to eliminate it, leading to persistence or dormancy, with no disease symptoms (12). Only a small fraction (about 10-15%) of LTBI positive individuals will re-activate to active disease, often several decades after initial exposure (13). Latency could have evolved as a strategy for the pathogen to ensure transmission and avoid extinction, in small host populations (14). Supporting this view, *M. tb* has evolved several mechanisms to survive during latency (15). Mathematical models suggest that optimal levels of persistence in human populations would be achieved with a latency period which is currently observed, suggesting that the human immune system keeps the latency and reactivation, under their evolutionary optimum (16). Thus, latency is neither a unique property of the host, nor that of the pathogen, but due to the interaction between both (17).

## Antagonistic relationship between IR and LTBI

Compared to active TB, LTBI in diabetes is poorly studied. Further, with respect to diabetes, most studies have been carried out only in chronic diabetes subjects, and what happens during the pre- and incipient of diabetes, is still an enigma (4, 18). Recently, we carried out LTBI screening, among individuals with various grades of increasing glucose intolerance (19). We found a decreased prevalence of LTB among pre-diabetes (23%) and newly diagnosed diabetes patients (23%), compared to healthy controls (33%) and chronic diabetes patients (33%) (19). It is important to note that gender plays an important role in disease susceptibility towards both diabetes as well as LTB. Male gender was a significant risk factor for both diabetes as well as LTBI (19). The exact reason for the decreased prevalence of LTBI among pre-diabetes subjects is not known.

Pre-diabetes is characterized by IR, which is the inability of the body to respond to insulin (20) (**Figure 1**). It is a clinical condition, characterized by hyperglycemia and hyperinsulinemia. Meta-inflammation during pre-diabetes induces IR, and to meet up with the increased insulin demand, pancreatic beta cells are coaxed to produce excess insulin. It is characterized by a unique cytokine profile, which includes both innate and adaptive immune cytokines (21, 22) (**Table 1**). Increased circulating levels of innate immune markers including: Type-I interferon (Interferon-β), pro-inflammatory cytokines (TNF-α, IL-1β and IL-6) and anti-inflammatory cytokines (IL-10, IL-1Ra and TGF-β) (19, 23, 24). TNF-α, IL-1β and IL-6 are known to activate macrophages and restrict mycobacterial growth (25). This in turn can either enable intracellular bacterial elimination or prevent LTB activation (25). IL-27 and IL-38 are recently discovered novel anti-inflammatory cytokines which belong to the IL-12 (26) and IL-1 (27) families, respectively. While IL-27 levels are increased, IL-38 are significantly reduced in pre-diabetes (19, 23, 24). With respect to adaptive immune cytokines, increased IL-17 and decreased IL-9 levels were noted, with no much changes in Th1 and Th2 cytokine levels (19, 23, 24). In contrast to pro-inflammatory cytokines, the main target cells for IL-17 are the neutrophils (28). Neutrophils dominate the first wave of innate immune response to *M. tb* infection (29). IL-17 activates neutrophils and promotes *M. tb* phagocytosis and expression of defensins (needed for intracellular killing) (30). It also induces IL-8 secretion which promotes neutrophil recruitment, to the lungs (30). Neutrophils possess two primary mechanisms of pathogen clearance: phagocytosis followed by intracellular killing and degranulation followed by extracellular killing**;** both have been implicated in protective immunity against *M. tb (* 31). Neutrophils utilize NADPH oxidase to produce reactive oxygen intermediates (ROI) inside their phagosome, for pathogen killing, as well as extracellular ROI, in response to soluble agonists (32). Compared to IL-17, the literature available on the role of IL-9 in *M. tb* infection is scant (33, 34). While IL-9 levels are lower in pre-diabetes, LTBI infection increases the level of this cytokine under conditions of co-morbidity (19, 23, 24). Augmentation of IL-9 during pre-diabetes might indicates better mucosal immune response against *M. tb* infection, and this interesting hypothesis needs further validation. *M. tb* infected macrophages are known to constitutively secrete high levels of IL-10 (35), which is known to reduce IR. LTBI during pre-diabetes, augments IL-10, IL-38, IL-9 and IL-12 and reduces TNF-α and IL-23 (19, 23, 24). These changes can either dampen meta-inflammation or augment anti TB immunity.

**Table 1:** Circulating levels of Immune Mediators in PDM, NDM and KDM.

Classification	Biomarkers	PDM	NDM	KDM
Type-1 Interferon	IFN-β	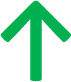	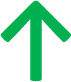	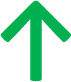
Pro-inflammatory cytokines	TNF-α	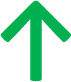	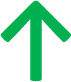	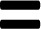
	IL-6	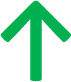	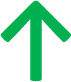	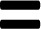
	IL-1β	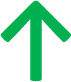	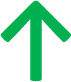	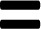
	IL-15	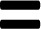	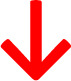	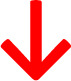
Anti-inflammatory cytokines	IL-1Ra	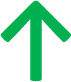	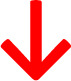	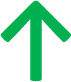
	IL-10	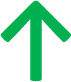	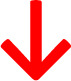	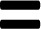
	TGF-β	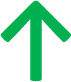	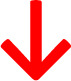	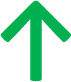
	IL-27	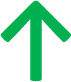	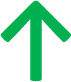	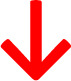
	IL-38	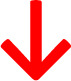	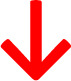	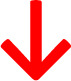
Adaptive immune cytokines	IL-12	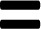	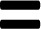	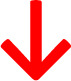
	IFN-γ	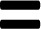	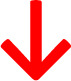	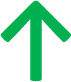
	IL-2	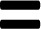	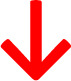	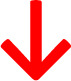
	IL-33	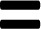	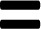	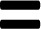
	IL-4	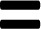	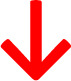	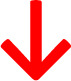
	IL-23	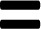	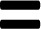	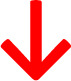
	IL-17	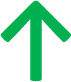	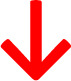	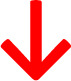
	IL-9	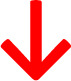	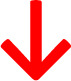	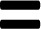
Chemokines	MCP-1	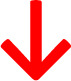	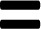	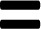
	RANTES	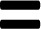	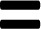	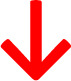
	IL-8	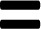	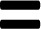	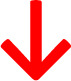
	IP-10	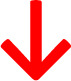	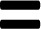	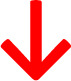
	SDF-1	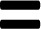	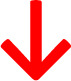	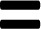
Defensins	α-Defensin	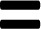	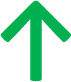	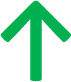

↑- Indicates higher levels compared to healthy controls.

↓- Indicates lower levels compared to healthy controls.

= Indicates no difference in the levels compared to healthy controls.

LTBI is known to upregulate adiponectin, leptin and FGF-21 in pre-diabetes individuals (19, 23, 24). While infiltration of *M. tb* infected macrophages into adipose tissue is the most likely possibility, direct infection of adipocytes with *M. tb* cannot be ruled out. In fact, direct adipocyte infection with *M. tb* is documented and this leads to the secretion of leptin, adiponectin and FGF-21 (36, 37). Adiponectin is a protective hormone against diabetes, since it promotes insulin sensitivity and also protects endothelium from redox damage (38). Interestingly, adiponectin is known to induce the secretion of IL-10 from macrophages (39, 40). The high levels of leptin seen during pre-diabetes could be due to the action of TNF-α (41) and IL-6 (42), on adipocytes. Leptin is known to act on macrophages and induce anti-mycobacterial immunity (43). FGF-21 is a recently discovered hormone which normalizes lipid metabolism and this can confer protection against both IR (44) and LTBI (45). Recently, IL-10 secreting macrophages were found to promote gastric epithelial healing and augment incretin secretion (46). Incretins are gut peptides, which at one end, can augment insulin sensitivity, and at the other end, protect against pancreatic beta-cell loss (47). This is indicated by high levels of incretins seen in TB patients (48). While circulating levels of these biomarkers (serum cytokine profile) can give interesting insights about the reciprocal relationship, deeper insights can be obtained only from cellular and molecular studies. Thus, a complex immune-endocrine network acts during the pre-diabetes stages which confers significant protection against LTBI. Alternatively, LTBI can orchestrate this immune-endocrine network conferring protection against IR.

## Macrophages as mediators of inverse relationship between LTBI and IR

**Figure 2:**
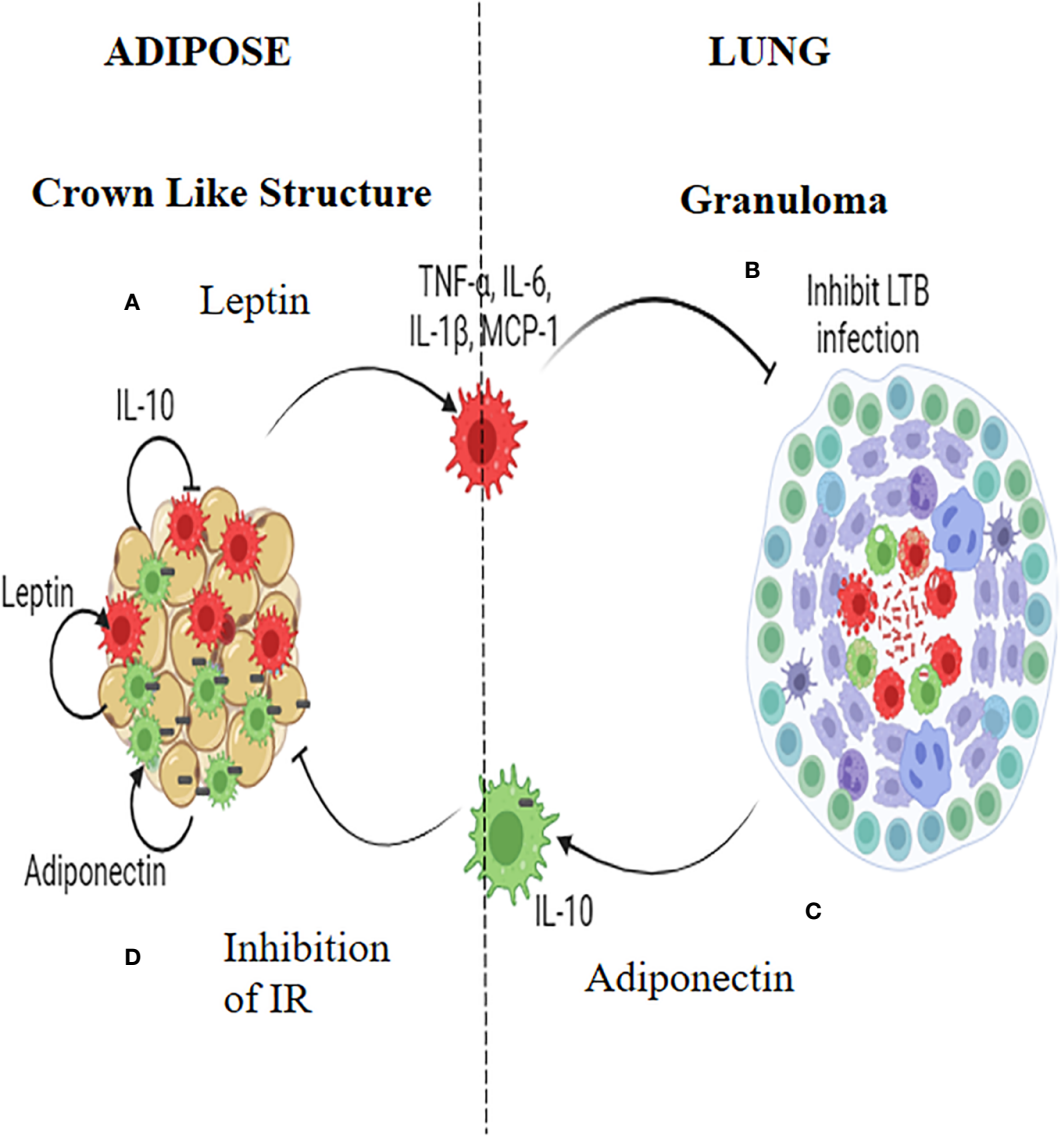
Insulin Resistance and Latent tuberculosis (IR-LTB) antagonism: In those who have IR, meta-inflammation in the adipose tissue leads to the polarization of pro-inflammatory macrophages, which constitutively secrete high levels of pro-inflammatory cytokines (TNF-α, IL-6 and IL-1β) and chemokine (MCP-1). This is mediated by leptin secreted by the adipocytes **(A)**. These macrophages enter the circulation, navigate into alveolar parenchyma, and restrict the growth of *Mycobacterium tuberculosis (M. tb)* **(B)**. In those who are LTBI, infection of alveolar macrophages with *M. tb*, brings about the polarization of anti-inflammatory macrophages which, constitutively secretes, high levels of IL-10 **(C)**. These AAMs enter the circulation, navigate to adipose tissue and attenuate meta-inflammation, there by conferring protection against IR. Alternatively these macrophages can act as Trojan horses transporting the *M. tb* into adipose tissue were in they can directly augment the secretion of adiponectin. Adiponectin can aid in the maintenance of the polarized state of these macrophages. Thus, macrophages serve as major mediators of IR-LTB antagonism **(D)**.

Macrophages seem to play a pivotal role, in mediating the inverse relationship between IR and LTBI (**Figure 2**). TNF-α, IL-6 and IL-1β which are abundantly secreted by adipocytes during IR, polarizes the adipose resident macrophages to a pro-inflammatory phenotype, which can have dual effect: They can restrict *M. tb* growth (or even eliminate the bacilli) when infected, but on the other end, in-flame meta-inflammation, worsening IR, in the adipose and liver (49). Alveolar macrophages, which are the first immune cells to get infected with *M. tb*, constitutively secrete high amounts of IL-10, which can have a dual effect: These cells can infiltrate adipose tissue and can dampen meta-inflammation, but on the other hand act as conducive reservoirs for LTBI (50). Alternatively *M. tb* infected macrophages can act as Trojan horses, transporting *M. tb* to adipose depots, were they can directly infect the adipocytes, inducing adiponectin secretion (50). IR facilitates trained immunity in macrophages conferring protection against TB (51, 52). However, the beneficial effects of IR on macrophages are lost during chronic diabetes condition (53). In an elegant study, conducted in the Mexican population, both monocytes and monocyte derived macrophages from diabetic patients, showed decreased expression of HLA-DR, CD86 and CD163 and increased expression of PD-L1 (54, 55). Further, upon infection with virulent *M. tb* strains, MDMs from diabetes patients showed changes in the expression of PD-L1. The secretion of cytokines (e.g., IL-6, IL-1β, IL-10, and IL-12) and chemokines (e.g., MCP-1, MIG, and RANTES) from these MDMs were also altered (55). In response to the more virulent *M. tb* strains, the levels of association and bacterial clearance were diminished in MDMs derived from diabetes patients (54).

Several *in vitro* experiments to demonstrate altered macrophage function under diabetic condition have largely yielded negative results since apart from hyperglycemia, several yet to be identified factors play a vital role in altering macrophage function under diabetic condition (56). For example, macrophage differentiated under hyperglycemic conditions were associated with marginal increase in cytokine production upon stimulation with *M. tb* lysate, with no differences in phagocytosis or intracellular killing *M. tb* (56).

## Inverse relationship between LTBI and incipient diabetes

In contrast to pre-diabetes, diabetes is characterized by hyperglycemia, insulin deficiency and IR (20) (**Figure 1**). It is interesting to note that, most immune biomarkers do not follow the parabolic curve, like that of blood glucose, with increasing glucose intolerance. Incipient diabetes, like pre-diabetes is characterized by increased circulating levels of innate immune markers which includes: Type-I interferon (Interferon-β), pro-inflammatory cytokines (TNF-α, IL-1β and IL-6) and anti-inflammatory cytokines (IL-10, IL-1Ra, TGF-β and IL-27), while IL-38 remain lower, like that of pre-diabetes (19, 23, 24). TGF-β, IL-1Ra and IL-15 levels drop abruptly while α-Defensin-1 peaks during pre-diabetes to diabetes transition (19, 23, 24) (**Table 1**). The increased levels of pro-inflammatory cytokines and decreased levels of anti-inflammatory cytokines, mark the transition between pre-diabetes to diabetes (19, 23, 24). With respect to adaptive immune cytokines, the increased levels of IL-17, seen during pre-diabetes, declines abruptly along with IL-2, IL-4, IFN-γ, while the IL-9 levels remain low (19, 23, 24). The acute deficiency of many of the adaptive immune cytokines marks the pre-diabetes to diabetes transition. The increased levels of defensins and decreased levels of adaptive immune cytokines are unique to pre-diabetes to diabetes transition (19, 23, 24). However, with respect to anti-TB immunity there is no much change between pre-diabetes and incipient diabetes (19, 23, 24). The prevalence of LTBI was identical under both conditions (19). The pro-inflammatory macrophages which are generated in the adipose tissue, can enter circulation and can confer significant protection against LTBI, while the anti-inflammatory IL-10 secreting alveolar macrophages, generated due to *M. tb* infection, can move to adipose tissue, and quench IR (49, 50).

## Synergistic interaction between chronic diabetes and tuberculosis (DM-TB synergy)

In contrast to incipient diabetes, chronic diabetes has a more complex cytokine profile (**Table 1**). Most of the pro- and anti-inflammatory cytokines including TNF-α, IL-6 and IL-1β decline and reach normal levels, with glycemic control and anti-diabetic treatment (19, 23, 24). However, with respect to anti-inflammatory cytokines, IL-10 reaches normal level, but TGF-β and IL-1Ra increases (19, 23, 24). Interferon-β and α-defensin-1 remain higher, while IL-15, IL-27, IL-38 levels are lower in chronic diabetes (19, 23, 24). With respect to adaptive immune cytokines, while IFN-γ levels are significantly increased, IL-9 levels which remain low during incipient diabetes increased and reaches normal values (19, 23, 24). However, IL-2, IL-4, IL-17, IL-12 and IL-23 are significantly lower in these patients (19, 23, 24). Out of all these cytokines, LTBI specifically downregulates IFN-β, IL-15, IL-1Ra, TGF-β, IL-12, IL-2, IL-33, IL-4, IL-9 and α-defensin-1 and upregulates IL-10, IL-27, IFN-γ, IL-17 and CRP (19, 23, 24). The significant downregulation of IL-12 and upregulation of IFN-γ indicates that other cytokines can augment Th1 polarization, in LTB+ chronic diabetes subjects (57). The low levels of both IL-33 and IL-4 indicate downregulation of Th2 response. Downregulation of IFN-β and α-defensin-1 indicates impaired anti-TB immunity (22).

The prevalence of LTBI among chronic diabetes cases doesn’t seem to be significantly higher, compared to that of control subjects (19). This is in striking contrast to the higher prevalence of active TB cases, among chronic diabetes (18). This apparent disparity between LTBI and TB seen among chronic diabetes patients, could be due to higher rate of reactivation of LTBI due to impaired anti-TB immunity, in these patients (compared to controls), compared to exogenous infection (58). The DM-TB synergy is due to a vicious cycle: The tubercle bacillus induces a strong inflammatory response and redox stress which can worsen IR and pancreatic beta-cell loss, precipitating in diabetes, among pre-diabetics, or worsen glucose intolerance, in chronic diabetics (59). On the other hand, chronic diabetes weakens the immune system, increasing the chances of TB infection, among uninfected (exogenous infection), or augmenting re-activation, in those with LTBI (endogenous reactivation) (3).

## T cells as mediators of DM-TB synergy

**Figure 3:**
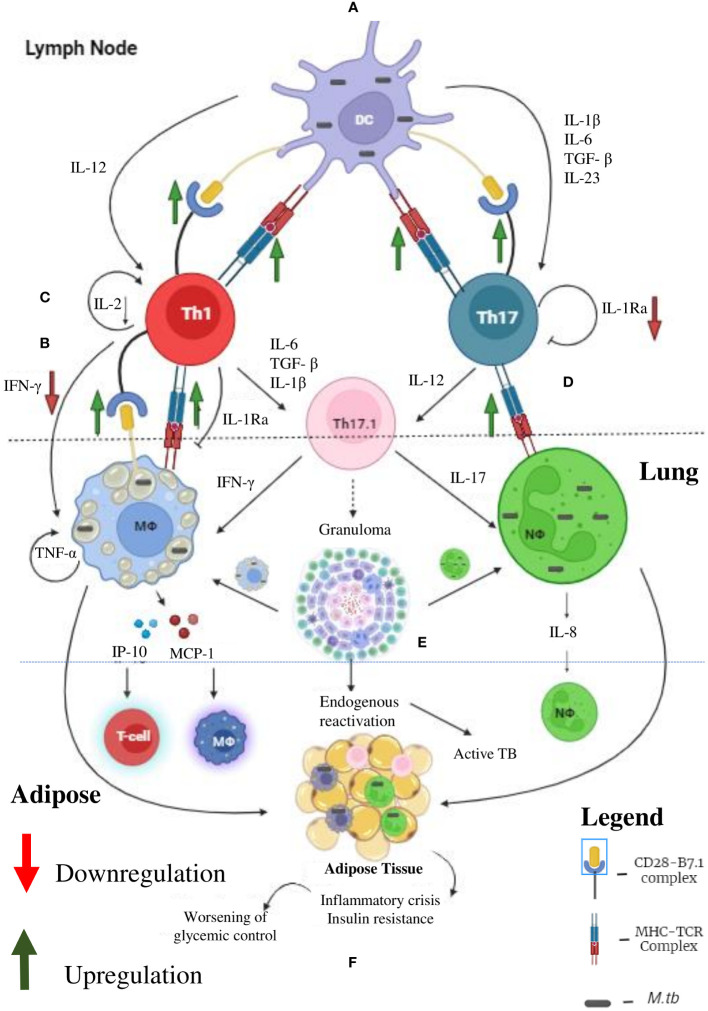
Tuberculosis (TB) and Diabetes (DM) Synergy: Under diabetic condition the professional antigen presenting cells like dendritic cells, macrophages and B cells (not shown) have significant upregulation of MHC and co-stimulatory (B-7) molecules, which leads to enhanced Th1 and Th17 polarization **(A)**. Decreased systemic levels of IL-12 and IL-23 leads to defective polarization. However, due to defective antigen induced secretion of IFN-γ, by the Th1 cells, defective T cell-Macrophage interaction takes place **(B)**. Further, due to defective antigen induced secretion of IL-2, the fitness of activated T cells is reduced **(C)**. Finally, due to defective antigen induced secretion of IL-1Ra, the Th1 and Th17 cells transdifferentiate into hybrid Th17.1 cells, which are pathogenic **(D)**. These defects leads to impaired acquired immune response which favors *M. tb* growth **(E)**. Further, IP-10 and MCP-10 secreted by *M. tb* infected macrophages attract T cell and monocytes, respectively. IL-8 secreted by neutrophils augment neutrophil influx. The inflammation and redox stress (not shown) induced during active TB disease worsens glycemic control **(F)**. Thus, helper T cells serve as major mediators of DM-TB synergy.

In contrast to macrophages, which plays an important role in LTBI during pre-diabetes and incipient diabetes, T cells seems to play a more vital role in LTBI, during chronic diabetes (60, 61) (**Figure 3**). Cytokine profiling in Quantiferon TB supernatants indicate several defects in cytokine secretion from *M. tb* specific T cells (19, 23, 24). Most importantly, *M. tb* antigen specific secretion of IFN-γ, IL-2 and IL-1Ra is significantly reduced, in chronic diabetes (19, 23, 24). IFN-γ along with TNF-α and IL-1β together form the main axis for anti-TB immunity (62). The reduced secretion of IFN-γ could be responsible for the poor detection of LTBI, among chronic diabetes subjects (63). Further, this observation also raises concern about the utility of IGRA, in detecting LTBI, in chronic diabetes subjects (63). Next to IFN-γ, IL-2 plays an important role in T cell activation. Activated T cells secrete high levels of IL-2 which binds to the upregulated IL-2R (CD25), forming an autocrine loop (64). This loop protects T cells from activation induced apoptosis and is needed for the complete activation of T cells (65). Defective secretion of IL-2 would render these T cells susceptible for apoptosis (66). Compared to IFN-γ and IL-2, the exact function of antigen specific secretion of IL-1Ra, in T cell biology is not known (67). At one end, it can inhibit the pathogenic action of excess IL-1β, secreted by *M. tb* infected macrophages (67). At the other end, it can inhibit the conversion of Th1 cells into the pathogenic Th17.1 cells, by the combined action of IL-1β and TGF-β (68). IL-1β and TGF-β are the major polarizing cytokines for Th17 differentiation. On the other hand, Th17 cells have extreme plasticity and have the ability to transdifferentiate into the pathogenic hybrid Th1-Th17 cells, under the action of IL-12 (69). Recently, these hybrid Th1-Th17 cells have been identified as major pathogenic players, in several autoimmune diseases (70). Further, upregulation of MHC and B-7 molecules in APCs (dendritic cells, B cells and macrophages) have been noted in chronic diabetic patients (71, 72). Upregulation of antigen presentation machinery, along with defective IL-1Ra secretion, indicates that, chronic diabetes might activate a dormant, pathogenic, autoreactive Th17.1 cells, which might promote LTBI reactivation and might also worsen active TB disease (73). Recently, pathogenic Th17.1 cells have been associated with poor prognosis in TB (68). However, whether chronic diabetes augments these cells needs to be tested. With respect to chemokines, MCP-1, IP-10 and IL-8 acts as a major chemoattract for macrophages, T cells and neutrophils. Defective secretion of these chemokines in chronic diabetes is noted which impairs anti-TB immunity.

## Other immune cells in IR-LTB antagonism and DM-TB synergy

In general, the current knowledge of altered immunity in DM patients with TB, indicates underperforming of innate immunity, but exaggerated adaptive immunity to M.tb, which includes various molecular mechanisms and pathways in the host (74). These include excess advanced glycation end products and their receptor (AGE/RAGE) signaling, oxidative stress, epigenetic changes due to chronic hyperglycemia, altered nuclear receptors, and alterations in leukocyte metabolism (immunometabolism) (74). Peripheral blood transcriptional signatures indicate enhanced inflammation, but attenuated type-I interferon responses, in DM-TB co-morbid individuals (75). The IR-LTB antagonism is a relatively new concept, compared to DM-TB synergy, and thus the involvement of various immune cells (apart from macrophages and T cells), is currently not known. Dendritic cells (DCs) in general play an important role in both IR and anti-TB immunity (76, 77). Infiltration of DCs into adipose tissue fuels meta-inflammation and IR (76). Activation of alveolar DCs with *M. tb* initiates the transition from innate to adaptive arm of anti-TB immunity (77). With respect to B cells and antibodies, infiltration of adipose tissue by B cells induce meta-inflammation and fuel IR (78). Interestingly, few studies have shown both beneficial (79) and harmful effect (80) of naturally occurring autoantibodies in IR. The role played by humoral immunity is TB is debatable. While most studies have shown neutral effect, few studies have shown beneficial effect for the pathogens (81). Like other immune cells, in obesity induced inflammation, neutrophils and basophils do infiltrate adipose tissue and fuel IR (82). However, infiltration of adipose tissue with eosinophils was found to confer significant protection against IR (83). Eosinophils are the major source of IL-4, which polarizes the macrophages into the alternative phenotype (83). This blunts IR. With respect to TB, neutrophilic response was shown to have both beneficial and harmful effect on *M. tb* clearance (84). IL-8 secreted by *M. tb* infected macrophages induce infiltration of mature CXCR1+CXCR2+ neutrophils (84). The infiltrated neutrophils phagocytose *M. tb* (84). But neutrophils in general are inefficient in killing *M. tb*, without T cell help. In presence of IL-17 (secreted by Th17 cells), neutrophils effectively clear the phagocytosed *M. tb* (84). In the absence of T cell help, neutrophils undergo *M. tb* induced necrosis exuberating inflammation (84). The role played by eosinophils and basophils, in TB infection is less clear. The involvement of newly discovered innate lymphoid cells (ILC-1,2 and3) in IR-LTB antagonism and DM-TB synergy is not known.

## Active TB versus latent TB

As mentioned previously, LTB is associated with immunomodulation and can confer protection against IR. However, the immunomodulatory effect associated with LTB is completely lost in active TB, which is associated with acute inflammation (85). Thus, the cytokine/chemokine profile changes completely during LTB to active TB conversion (85, 86) (**Table 2**). Most importantly, while LTB downregulates most of the pro-inflammatory cytokines like TNF-α, IL-6, IL-1β, active TB augments the secretion of these cytokines (87, 88). LTB downregulates IL-1Ra and upregulates IL-10, while in active TB downregulation of IL-1Ra and upregulation of IL-10 is seen (87, 89). A similar reciprocal pattern is also seen for other anti-inflammatory cytokines like IL-38 and IL-27 (90, 91). TGF-β is exceptional since high levels are seen both in LTB and active TB (92). With respect to adaptive immune cytokines LTB downregulates many of them while active TB augments their expression (92–94). A similar trend is also seen for chemokines (95–98)and defensins (98, 99).

**Table 2:** Circulating levels of immune mediators in DM-LTB and DM-ATB.

Classification	Biomarker	LTB	ATB
Type-1 Interferon	IFN-β	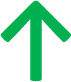	NA
Pro-inflammatory cytokines	TNF-α	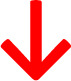	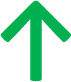
	IL-6	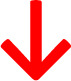	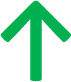
	IL-1β	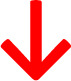	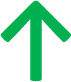
	IL-15	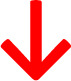	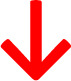
Anti-inflammatory cytokines	IL-1Ra	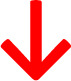	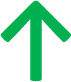
	IL-10	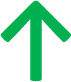	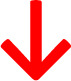
	TGF-β	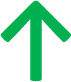	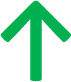
	IL-27	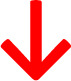	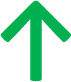
	IL-38	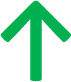	NA
Adaptive immune cytokines	IL-12	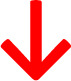	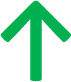
	IFN-γ	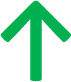	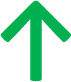
	IL-2	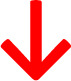	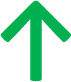
	IL-33	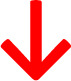	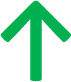
	IL-4	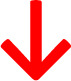	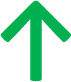
	IL-23	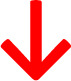	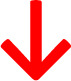
	IL-17	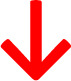	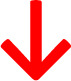
	IL-9	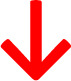	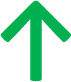
Chemokines	MCP-1	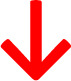	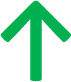
	RANTES	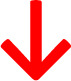	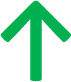
	IL-8	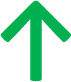	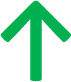
	IP-10	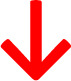	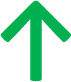
	SDF-1	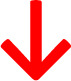	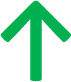
Defensins	α-Defensin	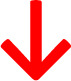	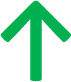
	β-Defensin	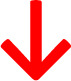	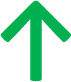
Hormones	Insulin	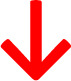	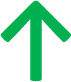
	Leptin	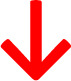	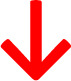
	Adiponectin	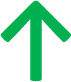	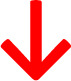
	FGF21	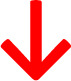	NA

↑- Indicates higher levels compared to healthy controls. ↓- Indicates lower levels compared to healthy controls. TB, Tuberculosis; LTBI, Latent Tuberculosis Infection; LTB , Latent Tuberculosis; ATB, Active Tuberculosis; IR, Insulin Resistance; DM, Diabetes Mellitus; M.tb, Mycobacterium tuberculosis; NADPH, Nicotinamide Adenine Dinucleotide Phosphate; IGRA, Interferon-Gamma Release Assay; ROI, Reactive Oxygen Intermediates; MHC, Major Histocompatibility Complex; HIV, Human Immunodeficiency Virus; LADA, Latent Autoimmune Diabetes in Adults; Th, T helper ; ILC, Innate Lymphoid Cells; DC, Dendritic Cells; BCG, Bacillus Calmette-Guerin; MDM, Monocyte Derived Macrophages; PD-L1, Programmed Death-Ligand 1; HLA-DR, Human Leukocyte Antigen-DR; APC, Antigen Presenting Cells; CD, Cluster of Differentiation; B7, B-lymphocyte activation antigen B7; MIG, Monokine induced by Interferon-gamma; IFN-γ, Interferon-γ; TNF-α, Tumor Necrosis Factor-α; IL, Interleukins; IL-1Ra, Interleukin-1 Receptor antagonist; FGF, Fibroblast Growth Factor; TGF-β, Transforming Growth Factor-β; MCP-1, Monocyte Chemoattractant Protein-1; RANTES, Regulated upon Activation Normal T cell Expressed and Secreted; IP-10, Interferon gamma induced Protein-10; SDF-1, Stromal Derived Factor-1; VEGF, Vascular Endothelial Growth Factor.

## Fighter gene hypothesis

**Figure 4:**
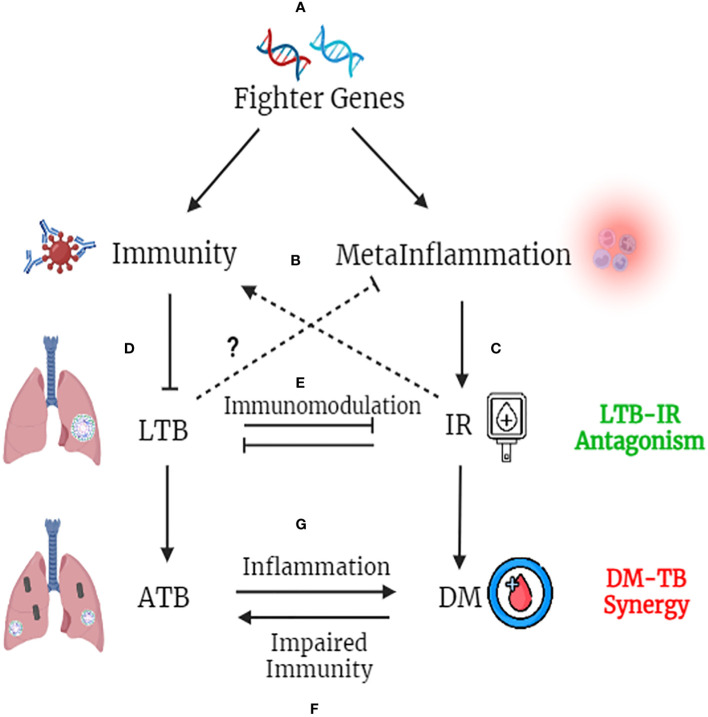
Fighter Gene Hypothesis: Both IR and LTBI have strong evolutionary history and have played a vital role in shaping the present-day human evolution. In tropical countries, were LTBI is high, polymorphisms in immune genes, which augment their activity, confers a selective advantage **(A)**. These “fighter genes” at one end confer superior anti-TB immunity, but at the other end, decrease the threshold for meta-inflammation **(B)**. Meta-inflammation leads to IR **(C)**. IR and immunity together confer protection against LTBI **(D)**. On the other hand, LTBI by means of immunomodulation, confers protection against IR, completing the LTB-IR antagonism loop **(E)**. However, if IR precipitates into diabetes (due to insulin deficiency), chronic glycemia impairs anti-TB immunity and promotes endogenous reactivation of LTB or exogenous *M. tb* infection **(F)**. Similarly, if LTBI precipitates into active TB, the acute inflammation and redox stress induced, will inflame meta-inflammation in adipose tissue, worsening the glycemic control, completing the DM-TB synergy loop **(G)**.

Global epidemiological studies have shown significantly high prevalence of LTBI in Asian and African countries (100). In these endemic zones, immune genes which confer better immunity would provide a selective advantage, which we propose as “Fighter Gene Hypothesis” (**Figure 4**). These gain-of function polymorphisms in immune genes, can confer chronic immunity, but augment meta-inflammation (101). The concept of better immunity packed with high inflammatory load is not new, and was first put forth by John Barthelow Classen more than decade ago (102). However, he was not able to explain the origin of this inflammation and hence calls it as “Iatrogenic Inflammation”, which increases the risk of type-2 diabetes but decreases the risk of type-1 diabetes (102). Meta-inflammation leads to IR. Both immunity and IR act synergistically in conferring protection against LTB (51). Interestingly insulin receptor deficient macrophages showed M2 phenotype (103) and conferred significant protection against IR (104), atherosclerosis (105) and skin inflammation (106) (**Figure 4**). Whether, the same insulin receptor deficient, M2 like macrophages, can confer protection against tuberculosis, is not known.

Similarly LTBI, has been shown to have strong immunomodulatory effect, which in turn, can confer protection against IR (107). However, with urbanization (abundance of carbohydrate rich food, lack of physical activity and chronic stress) increased insulin demand leads to insulin deficiency, which along with IR precipitates into diabetes, even in LTBI subjects (108). Further external factors like smoking, alcohol, air pollution, HIV infection etc. can weaken the immune system and can promote the conversion of LTBI to active TB, even in those with IR (58) (**Figure 4**). Unlike IR, diabetes weakens immunity and can promote the endogenous activation of LTBI leading to active TB (4). It can also make individuals more susceptible for new infections (4). Irrespective of whether it is endogenous reactivation or exogenous reinfection, diabetes co-morbidity worsens the outcome in TB (4). Similarly, active TB on the other hand, can worsen glycemic control in diabetes, completing the vicious cycle (109). Thus, the “IR-LTB Antagonism”, gets converted into “Diabetes-Tuberculosis synergy” due to the involvement of other factors (4).

## BCG vaccination and its effect on insulin resistance and type-2 diabetes

Neonatal BCG vaccination has sometimes been held responsible for inducing meta-inflammation and thereby increasing the risk for type-2 diabetes (102). However, till date no major epidemiological studies have been carried out to assess the effect of BCG vaccination on type-2 diabetes. Whether BCG vaccination can confer susceptibility or protection against type-2 diabetes is not clearly known. However, in a recent retrospective study conducted in the Canadian population, a small but significant protective effect was seen between BCG vaccination, against type-2 diabetes (but not LADA) (110). Even in animal models, a single dose of BCG was found to improvise glycemic control in diabetic rats (111). In these aspects, BCG acts like LTB conferring protection against type-2 diabetes. However, large epidemiological studies are needed to affirmatively ascertain the beneficial effect of BCG vaccination on type-2 diabetes.

## Conclusion

The interaction between TB and diabetes is not straight forward but multi-dimensional, and has a strong evolutionary history (112, 113). Both IR and TB infections, have played a vital role in shaping the evolution of humans (112, 113). While the former shapes metabolic evolution, the latter shapes immune evolution (114). IR, is a highly conserved phenomenon, from worms to humans (115). Unlike diabetes, IR provides several survival advantages, including better immunity (113). Intermittent fasting is known to induce both IR (116) and immunity (117), indicating a strong link between them. The beneficial effect of IR is lost once IR precipitates into frank diabetes. Similarly, the beneficial effect of LTBI is lost when it gets converted to active TB. Thus, the IR-LTB antagonism gets converted to DM-TB synergy due to both genetic and environmental factors. Obviously, this interesting bi-phasic phenomenon needs more investigation.

## Author contributions

VA: Conceptualization, Data curation, Formal Analysis, Funding acquisition, Investigation, Methodology, Project administration, Resources, Software, Supervision, Validation, Visualization, Writing – original draft, Writing – review & editing. SY: Conceptualization, Investigation, Software, Writing – review & editing.
